# Therapeutic Effect of Melatonin in Premature Ovarian Insufficiency: Hippo Pathway Is Involved

**DOI:** 10.1155/2022/3425877

**Published:** 2022-08-16

**Authors:** Ming Kang Qi, Tie Cheng Sun, Li Ya Yang, Jia Lin He, Yi Ming Guo, Han Bi Wang, Hui Ping Wang

**Affiliations:** ^1^Graduate School of Peking Union Medical College, Chinese Academy of Medical Sciences, Beijing 100730, China; ^2^NHC Key Laboratory of Reproductive Health Engineering Technology Research (NRIFP), National Research Institute for Family Planning, Beijing 100081, China; ^3^Reproductive Medical Center, Department of Obstetrics and Gynecology, Peking University International Hospital, Beijing 102206, China; ^4^Department of Obstetrics & Gynecology, National Clinical Research Center for Obstetric & Gynecologic Diseases, State Key Laboratory of Complex Severe and Rare Diseases, Peking Union Medical College Hospital, Chinese Academy of Medical Science and Peking Union Medical College, Beijing 100730, China

## Abstract

**Objective:**

Premature ovarian insufficiency (POI) is a female reproductive disorder of unknown etiology with no definite pathogenesis. Melatonin (MT) is an endogenous hormone synthesized mainly by pineal cells and has strong endogenous effects in regulating ovarian function. To systematically explore the pharmacological mechanism of MT on POI therapy, a literature review approach was conducted at the signaling pathways level.

**Methods:**

Relevant literatures were searched and downloaded from databases, including PubMed and China National Knowledge Infrastructure, using the keywords “premature ovarian insufficiency,” “Hippo signaling pathways,” and “melatonin.” The search criteria were from 2010 to 2022. Text mining was also performed.

**Results:**

MT is involved in the regulation of Hippo signaling pathway in a variety of modes and has been correlated with ovarian function.

**Conclusions:**

The purpose of this review is to summarize the research progress of Hippo signaling pathways and significance of MT in POI, the potential crosstalk between MT and Hippo signaling pathways, and the prospective therapy.

## 1. Introduction

Ovarian aging is a complex physiological process with multiple factors interacting and gradually accumulating, the essence of which is the decrease in the number and quality of follicles in the ovary, which is reflected in the reduction of reproductive and endocrine functions until they are lost [1]. However, premature ovarian insufficiency (POI) is a condition in which ovarian function is reduced or even fails before the age of 40 years, characterized by increased serum follicle stimulating hormone (FSH) and luteinizing hormone (LH) concentrations and decreased estradiol (E2) concentrations [2]. POI can lead to infertility, inadequate production of estrogen levels. It has a serious impact on women's physical and mental health, accelerating the onset of menopause. However, since POI is typically a heterogeneous disease, it is mainly treated with hormone replacement therapy. Till now, there is no clear and effective clinical treatment to restore or protect ovarian function [3, 4].

The Hippo signaling pathway is currently a hot topic among researchers. Studies have shown that the Hippo/Yes-associated protein (YAP) signaling pathway can regulate ovarian cell aging. For example, human umbilical cord mesenchymal stem cell-derived exosomes improve ovarian function and proliferation of POI by regulating the Hippo signaling pathway [5]. Even though we lack a complete understanding of the Hippo pathway, some studies have suggested that the Hippo signaling pathway may be involved in follicular maturation [6]. By manipulating the expression of key genes in the Hippo pathway in POI patients, Kawamura and colleagues successfully promoted follicle growth, recovered mature oocytes, and performed in vitro fertilization of the resulting oocytes [7]. This translates our understanding of the Hippo pathway into clinical applications and allows us to explore other roles that the Hippo pathway plays in ovarian function and disease.

Melatonin (MT), commonly known as a sleep hormone, controls physiological processes such as regulation of sleep-wake rhythms, body temperature, and physiological activity in circadian rhythms. MT also regulates ovarian function and is associated with the Hippo signaling pathway [8]. Furthermore, both Hippo signaling and melatonin are key regulators in neuronal differentiation of neuronal progenitor cells. Recently, emerging evidences illustrate the possible interaction between melatonin and Hippo signaling in different cell lines [9].

Despite recent advances in the field of reproductive endocrinology, the pathogenesis of POI remains unclear, which limits the development of POI treatment and has become a hot spot for researchers to gather [10, 11]. What is the relationship between the Hippo signaling pathway, MT, and POI, which are closely related to the regulation of ovarian function? Therefore, the authors reviewed the relevant studies on POI and tried to understand the pathogenesis of POI from the perspective of the Hippo signaling pathway [12, 13]. In addition, the authors also attempted to use MT to link the Hippo signaling pathway to POI, providing new ideas for the subsequent development of POI drugs.

## 2. Premature Ovarian Insufficiency (POI)

**Figure 1:**
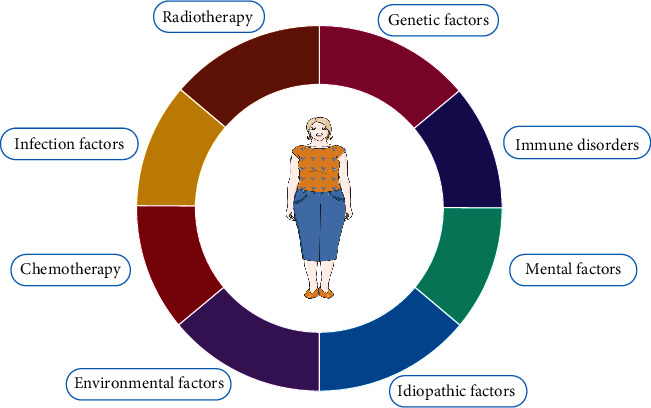
A variety of factors can reduce the number and quality of follicles, leading to ovarian dysfunction and ovarian aging, and ultimately POI [23].

For various reasons, POI is a condition in which ovarian failure occurs after menarche and before the age of 40 due to the destruction or depletion of follicles in the ovaries, causing a range of symptoms such as menstrual disorders and loss of libido [14]. POI can be diagnosed when a woman who is younger than 40 years old presents with menopause or sporadic menstruation for 4 months and has two consecutive FSH>25 U/L more than 4 weeks apart [15]. POI may be associated with genetic factors, immune disorders, iatrogenic factors of radiotherapy and chemotherapy, infection, and mental, environmental, and idiopathic factors, and its pathogenesis cannot be explained by a single factor or a certain gene [16] (Figure 1).

The incidence of POI in the general population of women is approximately 1-2% and a Swedish survey shows an increasing trend in the prevalence of POI in recent years [17]. The large population base of China has been seen a dramatic increase in the group of menopausal women, which was only 0.7 billion in 1982 and is expected to exceed 280 million in 2030 [18]. In addition, radiotherapy and chemotherapy can cause ovarian insufficiency and the incidence of POI increases year by year with the improvement of the survival rate of cancer patients [19].

Patients with POI can suffer from a range of symptoms due to low estrogen, such as menstrual cycle disorders or even amenorrhea; hot flashes; and night sweats, irritability, and low libido, as well as symptoms of perimenopausal syndrome such as osteoporosis, and reduced fertility or even infertility which happened to some patients [20, 21]. Also, the long-term effects of losing ovarian function too early include a higher risk of weak bones, heart disease, and problems with thinking and memory. This can even lead to metabolic dysfunction and a higher risk of death in women [12].

Follicles are the structural units of the ovary, comprising a single oocyte surrounded by supporting somatic cells. Folliculogenesis starts in fetal life with primordial follicle development, the majority of which will remain dormant. Folliculogenesis is a complex phenomenon that starts with the initial recruitment and activation of a primordial follicle. After activation, primordial follicles grow and mature into primary, secondary, and finally antral follicles. The mechanism underlying the developmental progression of human primordial follicles is unclear. However, the initial recruitment has been linked to protein kinase B (Akt) and mammalian target of rapamycin (mTOR) signaling pathways [6, 22].

Folliculogenesis and ovulation in normal ovary differ from that in POI ovary (impaired folliculogenesis). Under the regulation of intraovarian factors and gonadotropins, primary follicles develop into pre-antral and early antral follicles, which are the most susceptible to atresia, or follicle death. Then, they become preovulatory follicles, resulting in oocyte release and corpora lutea formation. Defects in folliculogenesis (e.g., decrease in primordial follicles, increase in atresia, and altered follicular maturation) cause POI [23]. Most of antral follicles in patients with POI are histologically abnormal [24].

Anti-Müllerian hormone (AMH) belongs to transforming growth factor beta superfamily. AMH expressed by pre-antral and small follicles granolosa cell is suggested by recent studies the best biomarker of ovarian reserve currently available [25]. With increasing age and size of follicle, the concentration of AMH has diminished. The most important role of this is prevention of further recruitment of other follicles during follicular development [26, 27]. For effective evaluation of the ovarian reserve, assessment of AMH is a good and helpful test [28, 29]. Its level in circulation is significantly correlated with the number of primordial follicles in healthy women [30].

The detectability of serum AMH in POI patients could be remarkably related to the presence of 15 or more follicles in their ovaries [24, 31, 32]. Although the number of cases in each group was not statistically sufficient, the mean serum AMH level was 2.16 ng/ml in women with 15 or more follicles and 0.42 and 0.33 ng/ml in women without follicles and those with five or fewer follicles, respectively. Although ovarian follicles are not visible on ultrasonography, assessing serum AMH could screen POI patients who are more likely to possess follicles that can eventually grow [31, 32].

The main mechanisms of POI pathogenesis like oxidative stress and inflammation are related to phosphatidylinositol 3-kinase (PI3K)/Akt pathway. Necrosis and necroptosis are involved in germ cell depletion from the mammalian ovarian cohort [33]. Oxidative stress and cytokines induce necrosis and necroptosis in the mammalian oocyte. Also, high levels of cytokines and oxidative stress induce necrosis and necroptosis in granulosa cells, resulting in follicular atresia. In granulosa cells, necrosis, as well as apoptosis, increases with the progression of follicular atresia [34]. Follicular rupture is considered an inflammatory response, and IL-1 and TNF-*α* are the major cytokines involved in this process [35, 36].

In the PI3K/Akt signaling pathway, once Akt is activated, it causes further cascades in the signaling pathway. Phosphorylated Akt phosphorylates a range of downstream target proteins, including forkhead box O 3 (FOXO3, anti-proliferative and apoptotic), Bcl-2-associated death promoter (BAD, a pro-regulatory member of the Bcl-2 family involved in the mitochondrial pathway), mTOR (controls protein biosynthesis and regulates cell growth), and p27 (maintains primary follicular stores) [37–39]. However, research in signaling pathways has identified the roles of Hippo signaling disruption and Akt stimulation of ovarian follicles for infertility treatment [7]. Inhibition of mitogen-activated protein kinases (MAPK) and PI3K/Akt signaling pathways can have a striking therapeutic effect on resection-induced POI in rats [40]. Furthermore, Hedgehog (Hh) signaling induces the Hippo pathway effector Yorkie (Yki) to promote the proliferation and maintenance of somatic follicular stem cells. Hedgehog and Yorkie pathways are coupled to regulate somatic stem cells (FSCs) proliferation but they act independently in escort cells (ECs), adjacent quiescent FSC derivatives, to limit bone morphogenetic protein (BMP) production and permit germline cell differentiation [41]. Huang's work demonstrates that an FSC lacking Yki or Hh pathway activity has reduced proliferation and is rapidly lost, whereas an FSC with excessive Yki or Hh pathway activity has an increased proliferation rate, extended longevity, and frequently duplicates and outcompetes wild-type FSCs in the same germarium [42].

POI diagnosis and treatment guidelines suggest hormone replacement therapy (HRT) as the main treatment, as well as stem cell therapy and traditional Chinese medicine [15]. Comprehensive lifestyle adjustment and health management are also recommended. The various treatment strategies described above are effective in relieving symptoms, but there are significant limitations among them. The administration of HRT, for example, has essentially no therapeutic effect on infertility due to POI and increases the risk of breast cancer, thrombotic disease. The WHI study results suggested a breast cancer increase in HRT users [Hazard ratio (HR) 1.26, Confidence interval (CI) 1.00–1.59]. This risk, in absolute terms, corresponds to 9 additional breast cancers per 10,000 women using estrogen-progestin therapy for five or more years [43, 44]. Stem cell therapy and traditional Chinese medicine approaches suffer from uncertainty of efficacy and lack of proven data from large-scale studies as well. In most cases, MSC therapy was quite efficient. However, the potential risk of MSC transplantation should be considered in terms of the long-lasting observations. Numerous reports both from in vitro and in vivo provided the evidence about MSC differentiation into certain cell types [45]. Current traditional Chinese medicine research technology is not designed to evaluate responses from multidimensional variables, like the herbal formulations used in TCM. This may be one of the reasons why the curative effects of TCM have not gotten approval among Western medicinal practitioners. New research techniques and methodologies should be developed to evaluate the curative effects of TCM and to elucidate its mechanisms [46, 47]. Therefore, understanding the etiology and pathogenesis of POI, with prevention and intervention of it, has become an urgent need of the current society.

## 3. There Is a Strong Connection between Melatonin (MT) and POI

### 3.1. MT Participates in the Normal Physiological Function of the Ovary

MT can delay ovarian aging, regulate ovarian biorhythm, promote follicle formation, and improve oocyte quality and fertilization rate [48]. As a free radical scavenger in the ovarian follicles, MT contributes to oocyte maturation, embryo development, and luteinization of granulosa cells [49, 50]. Besides, MT distinctly reduces the level of reactive oxygen species in oocytes, improves oxidative stress in oocytes, reduces early oocyte apoptosis, repairs mitochondrial integrity, improves spindle assembly and chromosome arrangement, and promotes meiotic maturation [51]. Moreover, MT administration delays ovary aging and improves fertility in mice via melatonin receptor type 1 (MT1)/AMP-activated protein kinase (AMPK) pathway [52, 53]. AMPK activation has been found to boost overall health and protect cells from oxidative stress-induced senescence [54]. A striking upregulation of AMPK and p-AMPK in the ovary of MT-treated mice was observed. Research has shown that the administration of melatonin can preserve ovary function and improve oocytes quantity and quality resulting in larger litter size compared to naturally aging mice at 24, 32,40, and 48 weeks old, respectively [52].

**Figure 2:**
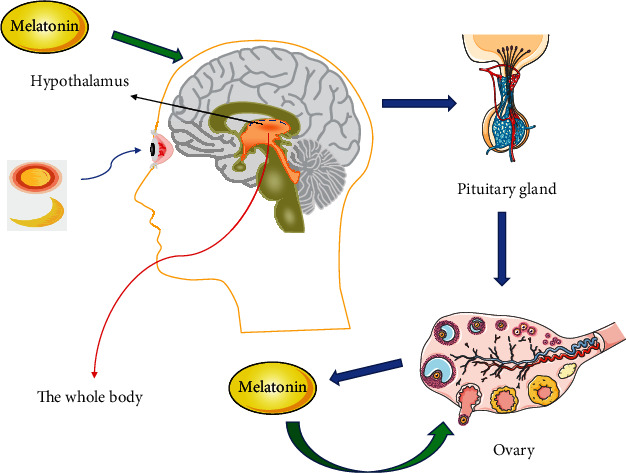
Melatonin (MT) secretory pathways and acting organs in the human body. MT can directly act on ovarian granulosa cells or indirectly act on ovarian granulosa cells through hypothalamic-pituitary-ovarian axis (HPO), thereby reducing the level of reactive oxygen species in oocytes and improving their oxidative stress state [51].

What's more, MT can act on the hypothalamic-pituitary-ovarian axis (HPO) via hypothalamic gonadotropins or directly bind to ovarian granulosa cells to exert effects on HPO [55]. Additionally, MT plays a central role in the reproductive system by upregulating LH receptor mRNA to suppress gonadotropin-releasing hormone (GnRH) and GnRH receptor expression as well [56]. As for the resource, MT in the ovary can be derived from systemic blood circulation or synthesized by granulosa cells, including the cumulus granulosa cells and oocytes [57–60]. MT1 and melatonin receptor type 2 (MT2) mRNAs can be detected in human granulosa cells and luteal cells [52]. During follicular development, MT concentrations in large follicles are significantly higher than those in small follicles. Before ovulation, MT concentrations in follicles are higher than those in serum, suggesting that MT plays an important role in follicular development and ovulation [61, 62] (Figure 2).

### 3.2. MT Delays Ovarian Aging

It has been found that long-term application of MT can slow down ovarian aging. MT delays ovarian aging through a variety of mechanisms, including antioxidation, DNA repair, telomere maintenance, silent information regulator (SIRT) family activity, ribosomal function, and autophagy [61, 63]. SIRT proteins are a class of proteins that possess nicotinamide adenine dinucleotide- (NAD+-) dependent deacetylase activity or adenosine diphosphate- (ADP-) ribosyltransferase activity. In mammals, seven SIRT proteins have been identified, from SIRT1 to SIRT7.

In the ovary, oocytes and somatic cells produce reactive oxygen species (ROS) and reactive nitrogen species (RNS) in the follicular microenvironment. These oxidants regulate the molecular and biochemical pathways involved in follicle formation, thereby destroying oocytes and causing follicular atresia [57, 60]. Oxidative stress can damage oocytes, granulosa cells, and mesenchymal cells in the ovary, thereby accelerating ovarian failure and possibly leading to malformations in embryonic development [64, 65]. These changes enhance apoptosis during pregnancy and weaken female fertility. On the other side, MT can reduce oxidative stress in a variety of ways and eliminate endogenous ROS and RNS, including the superoxide anions (O2·−), hydroxyl radicals, hydrogen peroxide (H2O2), nitric oxide (NO·), and peroxy-nitric anion (ONOO−) [66–68]. Complementarily, MT in human follicular fluid is involved in the protection of granulosa cells and oocytes by scavenging ROS produced by follicles during maturation and ovulation, inducing the synthesis and activity of antioxidant enzymes and thus preventing the induction of mitochondrial apoptosis [57, 60].

A growing body of data suggests that MT mediates reproduction by interacting with MT1 and MT2 in the ovary [69, 70]. In bovine granulosa cells (GCs), melatonin receptors MT1 and MT2 were differentially located at the cell membrane, the cytoplasm, and nuclear membranes. Expression of melatonin receptors (measured by real time-PCR) behaved differentially at various doses (melatonin) and time intervals. The MT1 mRNA increased significantly in a time-dependent manner (48 h), whereas MT2 mRNA was associated with both time intervals and melatonin dose [70]. Long-term administration of MT obviously increased in fetal number, follicular pool, telomere length, and oocyte quantity and quality [63]. Binding of MT to MT1 and MT2 can decrease reactive oxygen species (ROS) levels, increase the activities of glutathione S-transferase (GST) and glutathione peroxidase (GPx), and inhibit the level of glutathione (GSH) and plasma selenium [55, 71, 72]. It has been identified that MT inhibits apoptosis and boosts the expression of MT2, superoxide dismutase (SOD), and GPx4, while antagonists of MT1 and MT2 block the protective effects on follicular atresia and porcine granulosa cells [73].

The ovarian reserve is constituted by the quality and quantity of the primordial follicles, which both decline with increasing age [74]. The number of growing follicles recruited from the primordial follicle pool reflects the number of primordial follicles. Since there is no serum marker that can directly measure the number of primordial follicles, a marker that reflects the number of growing follicles is currently the best proxy for the quantitative aspect of the ovarian reserve. Since AMH is expressed by growing follicles prior to FSH-dependent selection and has been shown to be detectable in circulation, serum AMH has taken momentum as a marker for ovarian function, particularly in the assessment of the quantitative aspect of the ovarian reserve [75].

**Figure 3:**
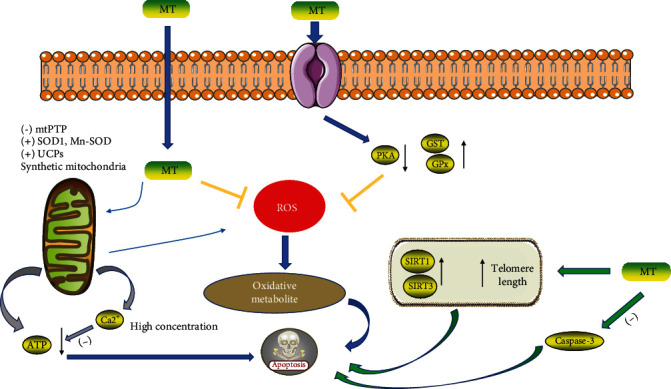
The mechanism of melatonin in resisting oxidative stress and delaying aging in cells. Abbreviations: MT: melatonin; ROS: reactive oxygen species; mtPTP: mitochondrial permeability transition pore; SOD: superoxide dismutase; Mn-SOD: Mn-superoxide dismutase; Ucp: uncoupling protein; GPx: glutathione peroxidase; PKA: protein kinase A; SIRT: silent information regulator [51].

The expression of silent information regulator family (SIRT1, SIRT3, and SIRT6, as well as Sirtuins) in the ovary is positively correlated with ovarian reserve [76]. These proteins may be potential markers of ovarian aging and target molecules for delaying organ aging. MT treatment can activate SIRT1 and SIRT3 mRNA expression in the ovary. It was shown that MT-induced upregulation of SIRT1 expression was associated with reduced oxidative stress, activation of antioxidant enzymes, and anti-apoptotic effects in mice and humans [77, 78]. Moreover, MT delays the senescence of mouse oocytes after ovulation through the SIRT1-MnSOD-dependent pathway [79]. Tamura found that mRNA expression of Sirtuins longevity genes (SIRT1 and SIRT3) and telomere length were also enhanced in melatonin-treated mice. MT could protect ovarian cells and slow down follicular atresia by activating SIRT1 and SIRT3 signaling [80] (Figure 3).

### 3.3. MT Regulates Ovarian Biological Rhythm

The biological clock system plays an essential role in the physiological activity of the ovary and is involved in the regulation of ovulation, steroid hormone synthesis, and oocyte maturation [81]. Disturbances in the biological clock can seriously affect ovarian function [81]. Ovarian granulosa cells and oocytes can secrete MT. Ovarian granulosa cells have MT receptors that play roles in the regulation of the ovarian clock. In fact, MT transmits photoperiodic messages that regulate reproductive activity, improve ovarian functions, and participate in the follicular development process, including ovulation [82, 83]. The disruption of circadian rhythms and altered light exposure due to human night work can negatively affect female reproduction at the molecular level, triggering an increase in infertility, menstrual disorders, and miscarriages [84]. In Finland, Kauppila observed a 2-hour extension of MT secretion in winter compared to summer [85]. During the dark season, the mean free testosterone levels and the free androgen index (FAI) were significantly decreased during the luteal phase of the cycle when the estradiol concentration was also decreased. A low estradiol concentration at the time of ovulation is a sign of diminished granulosa cell activity and suggests disordered follicular development. In a previous study from Finland, the decreased conception rate during the dark months correlated with day length but not temperature [86]. Seasonal environmental light modifies reproductive competence in humans, possibly via melatonin secretion [87].

## 4. Relationship between Hippo Signal Pathway and MT

**Figure 4:**
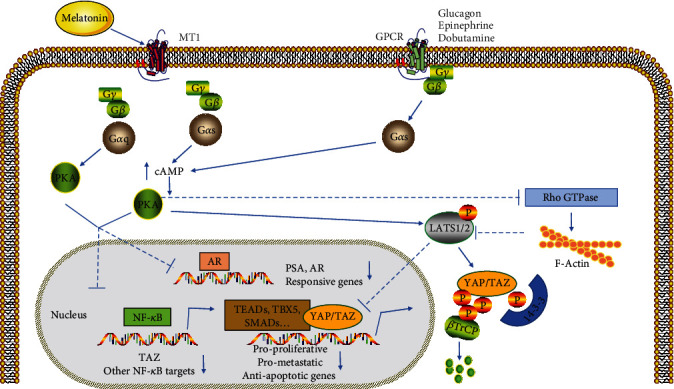
Interaction between melatonin and Yes-associated protein (YAP)/PDZ-binding motif (TAZ) signaling-regulated G-protein coupled receptor (GPCR) signals. ↑ indicates an increase in protein level or activity; ↓ indicates a decrease in protein level or activity. Abbreviations: MT1: melatonin receptor type 1; AR: androgen receptor; PKA: protein kinase A; NF-*κ*B: nuclear factor-*κ*B; LATS1/2: large tumor suppressor 1 and 2 [8].

As mentioned above, MT can signal through both MT1 and MT2 G-protein coupled receptors (GPCR). Activation of the receptors causes dissociation of the heterotrimeric G-proteins and the resulting G*α* subunit and G*βγ* complex interact with various effector molecules involved in cellular signaling [88]. Recently, GPCR signaling has been indicated to regulate the Hippo pathway [89] (Figure 4). Components of the Hippo pathway include membrane-associated proteins that sense cell polarity, cell density, and mechanical and metabolic signals that in turn activate a range of kinases, with the ultimate targets of the junctional proteins being the transcriptional co-activators YAP and PDZ-binding motifs (TAZ). Studies suggest that MT may promote the activation of YAP and TAZ through the regulation of the Hippo pathway [8]. Furthermore, MT attenuates cardiac reperfusion stress by improving optic atrophy 1- (OPA1-) related mitochondrial fusion in a Hippo/YAP pathway-dependent manner [90]. It is proved that there may be some sort of connection between MT and Hippo signaling pathway.

### 4.1. Gs Protein (G*α*s) May Be a Link between MT Signal and G-Protein Coupled Receptors (GPCR)/Yes-Associated Protein (YAP)/PDZ-Binding Motif (TAZ) Signal

YAP and transcriptional co-activators with TAZ are major targets of the Hippo signaling pathway and play important roles in the regulation of development, homeostasis, and regeneration [91–93]. GPCR signaling regulates the YAP/TAZ response to a variety of biochemical stimuli, and MT has been shown to activate G*α*s proteins associated with MT1 receptors in some cells and to increase intracellular cyclic adenosine monophosphate (cAMP) through subsequent activation of protein kinase A (PKA) and protein kinase C (PKC) [94–97]. Thus, G*α*s-mediated activation of PKA and PKC in response to different stimuli (adrenaline, melatonin, etc.) may inhibit cell proliferation and invasion through a series of convergent mechanisms, including large tumor suppressor 1 and 2 (LATS1/2) activation, inhibition of nuclear factor-*κ*B (NF-*κ*B) transcriptional activity, and suppression of the androgen receptor (AR) response in AR-positive cells [94, 98, 99]. Furthermore, according to a recent study, the TAZ promoter is directly targeted and activated by NF-*κ*B, suggesting that MT may potentially inhibit YAP/TAZ oncogenic function by increasing LATS1/2 activity (following PKA and PKC activation) or reducing TAZ transcription (following NF-*κ*B inhibition) [100].

### 4.2. The Relationship between YAP/TAZ and MT in Metabolic Pathways

It has been shown that the Hippo signaling pathway is closely related to the tissue metabolic pathway and they work together to regulate cell proliferation, differentiation, and apoptosis [101]. Glucose, fatty acids, hormones, and other metabolic factors have recently been illuminated to regulate YAP and TAZ, which also participate in metabolic regulation, such as promoting glycolysis, lipogenesis, and glutamine catabolism. YAP is known for its ability to positively regulate insulin and insulin-like growth factor-1 (IGF-1) signaling to drive insulin-like growth factor-2 (IGF-2) expression, activate mTOR signaling and AKT, promote glucose uptake and glycolysis, and drive growth advantage, metastatic capacity, angiogenesis, and therapeutic resistance in various model systems. Hence, it suggests that YAP and TAZ are emerging nodes that coordinate nutrient supply with cell growth and tissue homeostasis, and study on them may contribute to finding metabolic approaches to treat ovarian diseases [102, 103].

**Figure 5:**
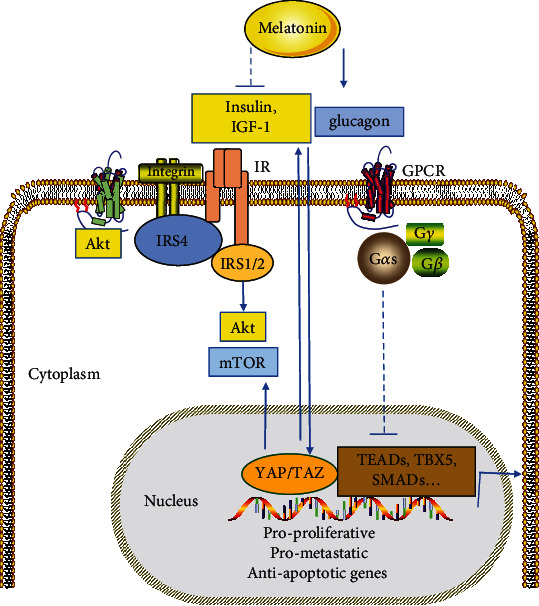
Interaction between melatonin, YAP/TAZ, and metabolic pathways. Abbreviations: IR: insulin receptor; IRS1/2/4: insulin receptor scaffold 1/2/4 [103].

In addition to GPCR signaling, YAP/TAZ is regulated by intercellular contact, mechanical force, and metabolism. They affect YAP/TAZ function by inducing specific intracellular signaling through Hippo kinase cascade-dependent and non-dependent mechanisms. Some of these mechanisms may be cross-linked with MT signaling. In conclusion, these evidences indicate that there may be crosstalk between MT signal, Hippo signal, and insulin-glucagon signal [101] (Figure 5).

### 4.3. Silent Information Regulator (SIRT) and Forkhead Box O (FOXO): Possible Links between MT Signals and Hippo Signals

Sirtuins, a family of NAD+-dependent deacetylases, have recently emerged as key metabolic sensors of body homeostasis. Together, they respond to metabolism, inflammatory signals, or oxidative stress, and have been linked to aging and longevity [104, 105]. The down-regulation of SIRT1 is associated with physiological or pathological reduction of ovarian reserve. SIRT1 has been proved to regulate the proliferation and apoptosis of granulosa cells, while there is hint that SIRT3 promotes luteinization [76]. Many transcription factors regulate SIRT1 expression, including forkhead box (FOXO) and peroxisome proliferator activated receptor (PPAR) [78].

Studies based on the SIRT1 activator SIRT1720 or resveratrol support the idea that targeting SIRT1 as a main factor in the regulation of follicular dynamics may be a promising strategy for the prevention of ovarian aging [106, 107]. The inhibition of Ras signaling by resveratrol counteracts Ras-PI3K-mediated protein kinase B activation, leading to increased FOXO3 activity. The fact that SIRT1 controls hemopoietic stem cell (HSC) homeostasis via FOXO3 further highlights the therapeutic value of SIRT1 activation [108].

SIRT1 coordinates the adaptive response of mouse oocytes to oxidative stress, possibly by promoting the activity of FOXO3 and SOD2 [77]. The SIRT1-dependent antioxidant response was disrupted in senescent oocytes, where a lower capacity to regulate SIRT1 expression was detected. Zhang reported that the SIRT1, 2, and 3 pathways may play a potential protective role in post-ovulatory oocyte senescence by controlling the production of reactive oxygen species [109]. It is pointed out that SIRT1 orchestrates the stress response of human granulosa cells to oxidative stress by targeting FOXL2, a transcription factor essential for ovarian function and maintenance [110, 111].

Aging and various age-related diseases are associated with reduced MT secretion, pro-inflammatory changes in the immune system, and reduced SIRT1 activity [112–114]. MT, in turn, is strongly associated with SIRT1. MT can act via stimulating or inhibiting components of the pro-inflammatory network. SIRT1 enhances the amplitude of circadian rhythms in the suprachiasmatic nucleus (SCN), which may affect MT rhythms [115, 116]. MT attenuates sepsis-induced brain damage through SIRT1 signaling activation [77, 117]. MT treatment significantly increases SIRT1 expression, leading to deacetylation of FOXO1 and p53. Melatonin can stimulate the release of pro-inflammatory cytokines and other mediators, but also, under different conditions, it can suppress inflammation-promoting processes such as NO release, activation of cyclooxygenase-2, inflammasome NLR family pyrin domain containing 3 (NLRP3), gasdermin D, toll-like receptor-4 and mTOR signaling, and cytokine release by senescence-associated secretory phenotype (SASP), and amyloid-*β* toxicity. It also activates processes in an anti-inflammatory network, in which SIRT1 activation, upregulation of nuclear factor erythroid-2-related factor 2 (Nrf2), down-regulation of NF-*κ*B, and release of the anti-inflammatory cytokines IL-4 and IL-10 are involved [118, 119].

The FOXO family of transcription factors (FOXO1, 3, 4, and 6) regulates the expression of genes associated with inhibition of cell cycle progression, hematopoietic differentiation, and resistance to stress [120]. FOXO3 has long been thought to play a key role in the molecular basis of longevity [121]. The ability of FOXOs to protect against oxidative stress involves increasing the expression of scavengers of reactive oxygen species (ROS), such as manganese superoxide dismutase (SOD2), catalase, and catalase reduction protein III in mitochondria. However, deacetylation of FOXO by members of the Sirtuin family 1, 2, and 3 leads to FOXO activation [122]. FOXOs promote the expression of genes encoding proteins related to DNA repair and inhibit the mTOR kinase pathway. Meanwhile, MT can enhance the activity of SIRT3, activate FoxO3 for nuclear translocation, and increase the binding of FoxO3 to the SOD2 promoter, leading to the trans-activation of antioxidant genes, consequently limiting the production of ROS in mitochondria and inhibiting mitochondrial oxidative damage [123]. In addition, YAP cooperates with TEA domain transcription factor (TEAD) to activate the expression of FOXD1, an age-protective protein. YAP deficiency results in the down-regulation of FOXD1. FOXO3 is a transcription factor that regulates the expression of core autophagy signaling genes, whose activities are strongly associated with the immune system dysfunction and neurodegeneration [124, 125]. It was assumed that melatonin abolished lipopolysaccharide (LPS) effects on autophagy impairment via regulating FOXO3 signaling [123].

Bonni demonstrated that the core factor Mst1 in the Hippo pathway can phosphorylate FOXO3 (followed by FOXO1), mainly Ser207 (Ser212 in FOXO1), which is a conserved site in the forkhead domain [126]. This phosphorylation prevents 14-3 binding and promotes FOXO nuclear retention and transcriptional activity. The positive regulation of FOXO1/3 by Mst1/Mst2 may be a physiological regulatory event, but the situation that causes Mst1/Mst2 to phosphorylate FOXO1/3 in vivo is not clear [127].

## 5. Correlation between Hippo Pathway and POI

Many molecules in the Hippo signaling pathway are in connection with the regulation of oocyte growth, primordial follicle development, and granulosa cell proliferation and differentiation [128]. Abnormal expression of various signaling molecules in the Hippo pathway may bring about deficiency in primordial follicle survival and development, resulting in a pathological state of the ovary and causing POI [129, 130]. Follicle activation can be achieved by mechanical manipulation of ovarian tissue cut into pieces [7]. Hippo signal disruption and Akt stimulation of ovarian follicles can be used in infertility treatment. After fragmentation of the ovary, the level of phosphorylated YAP was substantially reduced and the ratio to total YAP was significantly lower, indicating that the Hippo signaling pathway was disrupted. The Hippo signaling pathway plays a role in the control of follicle growth and oocyte maturation in the ovary [130].

Apart from follicular activation, the Hippo signaling pathway in POI has been barely studied, with only a few reports suggesting that it may be part of the mechanism of action of herbal or natural products. For example, Xie and colleagues demonstrated that the Huyang yangkun formula enhanced ovarian function in POI rats by repairing the dysfunction of the Hippo-JAK2/STAT3 signaling pathway [131]. Ai and colleagues found that Tripterygium glycosides promoted cytotoxicity, senescence, and apoptosis in ovarian granulosa cells by inducing endogenous miR-15a expression and inhibiting the Hippo-YAP/TAZ pathway [132].

The Hippo signaling pathway in granulosa cells plays an important role in regulating the proliferation and differentiation of granulosa cells induced by gonadotropin and acts as crucial factor in the growth and maturation of activated follicles [128]. Knockdown of YAP1 in ovarian granulosa cells driven by the FOXL2 promoter resulted in increased apoptosis, reduced number of corpus luteum, reduced ovarian volume, and reduced fertility in transgenic mice [133]. YAP1-mediated mechanisms can control cell survival and granulosa cell differentiation during ovulation [134].

For Hippo pathway genes, LATS1 deficiency in mice causes sterility and ovarian tumourigenesis, while LATS1 regulates the activity of FOXL2, which is a defective gene in some POI patients [135, 136].

The application of Hippo-specific inhibitors can provide strong evidence for the study of the correlation between follicular development and Hippo signaling pathway and provide guidance for the development of POI biological gene therapy or anti-POI drugs. However, the number and structure types of Hippo inhibitors reported are quite limited, and few clinical studies on the treatment of POI are conducted.

## 6. Conclusions and Perspectives

The pathogenesis of POI is complex and regulated by a combination of multiple factors and components. Numbers of studies on mammalian MT and Hippo signaling pathways have made progress in understanding the MT and Hippo action mechanism on POI (Table 1). There are no effective methods or proven drugs to prevent it. We attempted to elucidate the potential crosstalk between MT and Hippo signaling pathways, and the possible implications for POI therapy. Experimental data shows that MT and Hippo signaling pathways are jointly involved in follicle development, oocyte maturation, and granulosa cell proliferation and differentiation, which are critical for maintaining normal ovarian development and physiological functions. Moreover, MT and Hippo signaling pathways are cross-linked in the regulation of these functions. It means Hippo signaling pathway-related factors as well as MT signaling pathway-related factor targeting studies may be an interesting research direction for the treatment of POI [133] and that MT intervention therapy for patients may be a promising option. The development of POI involves multiple signaling pathways. Can the application of MT protect against POI by modulating the Hippo pathway? It remains to be explored how the Hippo signaling pathway interacts with MT and POI.

## Figures and Tables

**Table 1:** References concerning melatonin (MT) and Hippo signal improving POI.

	Animal	Design	Regulation	Year	Author/references
Granulosa cells	Mouse	Vivo, vitro, in vitro maturation (IVM)	Human umbilical cord-derived mesenchymal stem cell-derived exosomes (hUCMSC-exos) promoted granulosa cells (GCs) proliferation in vitro by regulating the hippo pathway and the effect was inhibited by a YAP inhibitor.	2021	Li ZK [5]
Follicle	Mouse	Vivo, vitro, IVM	Fragmentation of murine ovaries promoted actin polymerization and disrupted ovarian hippo signaling, decreased phospho-YAP (pYAP) levels, and increased nuclear localization of YAP, as well as enhanced expression of CCN growth factors and BIRC apoptosis inhibitors, promotion of follicle growth, and the generation of mature oocytes.	2013	Kawamura K [7]
Ovarian germline stem cell	Mouse	Vivo, vitro	Physiological and pathological ovarian aging mice showed decreased protein expression levels of the main hippo signaling molecules (pYAP1) and mouse vasa homolo (MVH)/octamer-binding transcription factor 4 (OCT4).	2019	Xu J [13]
Granulosa cells	Mouse	Vivo, vitro	fMSCs also upregulated MT1, JNK1, PCNA, and adenosine monophosphate kinase (AMPK) at the mRNA and protein levels.	2019	Huang B [48]
Ovarian senescence	Mouse	Vitro, IVM	SOD, catalase (CAT), silent information regulator 1 (SIRT1), Bcl-2↑, acetylated-forkhead box O1 (ac-FoxO1), acetylated-NF-*κ*B p65 (ac-NF-*κ*B), malondialdehyde (MDA), and Bax↓.	2015	Zhao L [77]
Ovarian tissues	Rats	Vivo, vitro	MT could protect follicular integrity; prevent cell apoptosis; decrease reactive oxygen species (ROS), MDA, and nitric oxide (NO) levels; and increase activities of glutathione peroxidases (GSH-Px), glutathione (GSH), CAT, and superoxide dismutase (SOD) in cryopreserved ovarian tissues (OTs).	2020	Sun TC [57]
Follicle	Mouse	Vitro	The antioxidant enzyme activities (including GSH-PX, GSH, SOD, and CAT) were enhanced and MDA content was significantly decreased.	2020	Liu XC [58]

## Data Availability

The datasets that were used or analyzed during the current study are available from the corresponding authors on reasonable request.

## Abbreviations

Ac-FoxO1: Acetylated-forkhead box O1
Ac-p53: Acetylated-p53
ADP: Adenosine diphosphate
Akt: Protein kinase B
AMPK: Adenosine monophosphate kinase
AR: Androgen receptor
BMP: Bone morphogenetic protein
cAMP: Cyclic adenosine monophosphate
CAT: Catalase
CI: Confidence interval
E2: Estradiol
FAI: Free androgen index
FOXO: Forkhead box O
FOXO3: Forkhead box O3
FSH: Follicle stimulating hormone
G*α*s: Gs protein
GCs: Granulosa cells
GnRH: Gonadotropin-releasing hormone
GPCR: G-protein coupled receptors
GPx: Glutathione peroxidase
GST: Glutathione S-transferase
GSH: Glutathione
GSH-Px: Glutathione peroxidases
Hh: Hedgehog
HPO: Hypothalamic-pituitary-ovarian
HR: Hazard ratio
HRT: Hormone replacement therapy
HSC: Hemopoietic stem cell
hUCMSC-Exos: Human umbilical cord-derived mesenchymal stem cell-derived exosomes
IGF-1: Insulin-like growth factor-1
IGF-2: Insulin-like growth factor-2
IVM: In vitro maturation
LATS1/2: Large tumor suppressor 1 and 2
LH: Luteinizing hormone
LPS: Lipopolysaccharide
MAPK: Mitogen-activated protein kinases
MDA: Malondialdehyde
MnSOD: Manganese superoxide dismutase
mTOR: Mammalian target of rapamycin
MT: Melatonin
MT1: Melatonin receptor type 1
MT2: Melatonin receptor type 2
MVH: Mouse vasa homolo
NF-*κ*B: Nuclear factor-*κ*B
NLRP3: NLR family pyrin domain containing 3
NO: Nitric oxide
Nrf2: Nuclear factor erythroid-2-related factor 2
OCT4: Octamer-binding transcription factor 4
OPA1: Optic atrophy 1
OTs: Ovarian tissues
PI3K: Phosphatidylinositol 3-kinase
PKA: Protein kinase A
PKC: Protein kinase C
POI: Premature ovarian insufficiency
PPAR: Peroxisome proliferator activated receptor
pYAP: Phospho-YAP
RNS: Reactive nitrogen species
ROS: Reactive oxygen species
SIRT1: Silent information regulator 1
SIRT3: Silent information regulator 3
SIRT6: Silent information regulator 6
SOD: Superoxide dismutase
TAZ: PDZ-binding motif
SCN: Suprachiasmatic nucleus
WHI: Women's health initiative
YAP: Yes-associated protein
Yki: Yorkie.
